# Diagnostic Value of Dynamic Magnetic Resonance Imaging (dMRI) of the Pelvic Floor in Genital Prolapses

**DOI:** 10.3390/biomedicines11102849

**Published:** 2023-10-20

**Authors:** Aida Gilyadova, Anton Ishchenko, Elena Puchkova, Elena Mershina, Viktor Petrovichev, Igor Reshetov

**Affiliations:** 1 Federal State Autonomous Educational Institution of Higher Education I.M. Sechenov First Moscow State Medical University of the Ministry of Health of the Russian Federation (Sechenov University), Ministry of Health of the Russian Federation, 119435 Moscow, Russia; reshetoviv@mail.ru; 2 National Medical Research Center Treatment and Rehabilitation Center of the Ministry of Health of the Russian Federation, 125367 Moscow, Russia; ra2001_2001@mail.ru (A.I.); petrovi4ev@gmail.com (V.P.); 3 Group of Companies “Mother and Child”, Clinical Hospital “Lapino”, 117209 Moscow, Russia; milleyk@mail.ru; 4 Medical Research and Education Center Moscow State University Named after M.V. Lomonsov, 119192 Moscow, Russia; elena_mershina@mail.ru

**Keywords:** pelvic organ prolapse, dynamic magnetic resonance imaging, diagnostic characteristics, T2-weighted sequences, multicompartment disorders, genital prolapse, pelvic floor muscle failure, vaginal wall prolapse

## Abstract

Pelvic organ prolapse is a chronic disease resulting from a weakening of the musculoskeletal apparatus of the pelvic organs. For the diagnosis of this pathology, it is insufficient to conduct only a clinical examination. An effective diagnostic tool is the method of dynamic magnetic resonance imaging (MRI) of the pelvic floor, which allows a comprehensive assessment of the anatomical and functional characteristics of the walls of the pelvis and pelvic organs. The aim of the study was to analyze the literature data on the possibilities and limitations of using dynamic MRI in pelvic organ prolapse. The widespread use of the dynamic MRI method is due to the high quality of the resulting image, good reproducibility, and the maximum ability to display the characteristics of the pelvic floor. Dynamic MRI of the small pelvis allows a comprehensive assessment of the anatomical and functional features of the pelvis, excluding the effect of ionizing radiation on the body. The method is characterized by good visualization with high resolution and excellent soft tissue contrast. The method allows for assessing the state of the evacuation function of visualized structures in dynamics. Simultaneous imaging of all three parts of the pelvic floor using dynamic MRI makes it possible to assess multicompartment disorders. The anatomical characteristics of the state of the pelvic organs in the norm and in the event of prolapse are considered. The technique for performing the method and the procedure for analyzing the resulting images are described. The possibilities of diagnosing a multicomponent lesion are considered, while it is noted that dynamic MRI of the pelvic organs provides visualization and functional analysis of all three parts of the pelvis and often allows the choice and correction of tactics for the surgical treatment of pelvic organ prolapse. It is noted that dynamic MRI is characterized by a high resolution of the obtained images, and the advantage of the method is the ability to detect functional changes accompanying the pathology of the pelvic floor.

## 1. Introduction

Pelvic organ prolapse and prolapse are a group of multifactorial diseases manifested by peripheral displacement of the uterus and/or vaginal walls in combination with adjacent pelvic organs (bladder, rectum, and bowel loops) beyond the vulvar ring as a result of functional failure of the ligamentous apparatus of the internal genitalia and pelvic floor muscles [1,2,3].

The prevalence of genital prolapse varies greatly according to various studies and ranges from 3 to 50% [2,4]. This variability is due to differences in the designs of the studies conducted, as well as the use of different approaches to the diagnosis of the conditions under study. In studies based on objective gynecological examination without taking into account subjective symptoms in the examined patients, the prevalence of pelvic organ prolapse (POP) reaches 50% [5].

Pelvic floor dysfunction occurs in 25% of non-pregnant women over 20 years of age in the population of economically developed countries, with 17% having moderate to severe urinary incontinence [6,7]. With increasing life expectancy, this pathology is expected to become particularly important in the near future. According to estimates by Wu et al. (2020), the proportion of women with symptomatic UTIs in the United States will increase to 46% by 2050 [6].

POP is divided into anterior compartment prolapse, which is represented by urethrocele, cystocele, and cystourethrocele; middle compartment prolapse, which includes uterine prolapse and vaginal vault prolapse; and posterior pelvic floor prolapse, which is represented by both rectocele and enterocele, depending on the impaired support of a particular component of the pelvic floor [8]. Anterior compartment prolapse is the most common among the above three types and is reported to be twice as common as posterior compartment prolapse and three times as common as apical prolapse [9].

Pelvic floor muscle incompetence with prolapse of the anterior vaginal wall manifests as symptoms such as urinary dysfunction, urinary incontinence, pelvic organ prolapse, dyspareunia, and pelvic pain, significantly impairing a woman’s quality of life.

dMRIs serve as valuable tools in the diagnosis of pelvic prolapse in complex cases involving multiple compartments and multiple organs of the pelvis. This method allows for a more accurate assessment of pelvic prolapse, as a higher resolution provides better anatomical detail compared to translabial ultrasound. To diagnose this pathology, it is not sufficient to perform only a clinical examination. An effective diagnostic tool is dynamic magnetic resonance imaging (dMRI) of the pelvic floor, which allows a comprehensive assessment of the anatomical and functional characteristics of the pelvic walls and pelvic organs [10,11,12,13,14].

Until recently, the standard approach in the examination of patients with POP was the assessment of the anterior pelvic floor based on the clinical examination, as well as using ultrasound examination (US) and urodynamic tests. However, in recent years, the dMRI method has been increasingly used due to the high quality of the image obtained, good reproducibility, and maximum ability to display pelvic floor characteristics [12,15,16]. Yet, despite its significant advantages, the literature continues to discuss the diagnostic accuracy of the method, searching for correlations between the data obtained with its help and the results of the clinical examination of the patient.

The results of pelvic dMRI are particularly important when the results of physical examination and combined urodynamic examination do not coincide. The data obtained can provide useful information during the pre- and postoperative examination of patients with POP undergoing surgical treatment, especially for multicompartmental abnormalities. dMRI makes it possible to conduct more effective preoperative planning in complex cases and select a more optimal method of surgical correction [17,18].

The purpose of the study was to analyze the literature data on the possibilities and limitations of the dynamic MRI method in POP.

## 2. Etiology and Pathogenesis of the Pathology of Pelvic Organ Support Structures

The etiological factors of pelvic floor dysfunction development include combinations of genetic, anatomical, physiological factors, high parity, and lifestyle factors that interact with each other in different periods of a woman’s life and lead to the occurrence of this pathology [19,20,21].

Neshatian L et al. conducted a retrospective study of patients over 18 years of age with genital prolapse at the Stanford Pelvic Health Center, the results of which indicate a correlation between the severity of pelvic organ prolapse and an increase in the proportion of fat in the lumbar muscles. Magnetic resonance defecography findings of pelvic floor weakness, pelvic organ prolapse, and rectal prolapse were characterized by standard MRD measurements and compared with the psoas (L4 level) and puborectalis muscle index, as well as relative fat fraction quantified using the 2-Dixon scoring method [12].

A study by DeLancey J.O. (2016) showed that under physiological conditions, pelvic support is provided by the interaction between the group of muscles that lift the anus and the ligaments that stabilize the vagina at different levels [22]. Any weakness or rupture of the connective tissue leads to pelvic floor defects. Three levels of connective tissue support of the vagina have been described [23]; with the cardinal and sacro-uterine ligaments providing the strongest support at the apical level [24,25]. However, changes in the structure of the extracellular matrix lead to pathological changes in the connective tissue, which eventually manifests as disorders of the function and integrity of the pelvic floor tissues and causes the development of prolapse [26].

## 3. Clinical Manifestations and Diagnosis of POP

Pathological changes in the anterior pelvic compartment affect the bladder, urethra and associated pelvic structures. Risk factors for this pathology include older age, a history of multiple pregnancies, a family history of pelvic floor disease, pelvic surgery and trauma, chronic increased abdominal pressure caused in particular by chronic constipation or chronic obstructive pulmonary disease, obesity, damage to nerves innervating the corresponding pelvic structures, connective tissue diseases such as Ehlers-Danlos syndrome.

Many women have symptomatic prolapse, which can affect their daily activities, sexual function, physical activity, and other aspects of quality of life. It is estimated that about 50% of women in labor have at least a mild degree of POP; the lifetime risk of undergoing pelvic reconstructive interventions in women is estimated at about 11%. Thus, this diagnosis is one of the most frequent indications for surgical treatment in women [27]. The most characteristic symptom is the sensation of a foreign body in the vagina extending beyond it. Patients with complete uterine prolapse may have puru-lent discharge from the vagina associated with the accession of secondary infection to the decubital ulcer. According to Lawrence J.M. et al. (2008), POP is often associated with the following diseases: 40% of patients suffer from urinary incontinence when straining, 37% suffer from overactive bladder and 50% suffer from fecal incontinence [28].

The combination of clinical manifestations resulting from pelvic floor disorders can cause psychological stress, which leads to decreased quality of life [29].

Urodynamic tests, pessary stress tests can help to diagnose the above-mentioned disorders in POP, but none of these tests can guarantee the accuracy of the diagnosis due to their very low sensitivity with regard to the prognosis of urinary incontinence (17–39%). On the other hand, although ultrasound has a high sensitivity in the diagnosis of stress urinary incontinence, this method does not have sufficient sensitivity for the diagnosis of prolapse [30].

dMRI of the pelvis allows a comprehensive assessment of the anatomical and functional features of the pelvis, excluding the exposure of the body to ionizing radiation [31]. The method is characterized by good visualization with high resolution and excellent soft tissue contrast. The method enables to assess the state of evacuatory function of visualized structures in dynamics [32].

Simultaneous visualization of all three regions of the pelvic floor using dMRI makes the assessment of multicompartmental abnormalities possible, allowing surgical treatment to be corrected in about 67% of cases [33,34].

## 4. Evaluation of Anatomical Characteristics in a dMRI

A clear understanding of the anatomy of the pelvic floor is an essential basis for accurate diagnosis of various pathological changes. When performing dMRI, the field of view includes the bladder, anterior urethra, uterus and cervix, vagina, rectum, and anal canal.

The main structures supporting the pelvic floor are the intrapelvic fascia, pelvic diaphragm, and urogenital diaphragm. Each of these structures has elements that support the bladder and urethra.

The intrapelvic fascia is a thin upper layer of fibromuscular connective tissue that covers the internal organs of the pelvis and the complex that raises the anus. This fascia consists of several components. The anterior part of the pubocervical fascia runs from the pubic bone to the cervix, providing support for the urethra and bladder neck. The three seals of the intravesical fascia are the urethral ligaments—the periurethral, paraurethral, and pubourethral ligaments. These structures also support the urethra and the bladder neck. The anterior vaginal wall and its attachments to the muscles raising the anus and to the pelvic tendon arch also provide support to the urethra and bladder, resembling a “loop” or “hammock” [35].

The parametrium and paracolpium are fascial extensions of the lateral walls of the pelvis that depart from the uterus and vagina. The parametrium forms seals that are uterosacral ligaments that support the uterus and the upper part of the vagina. The posterior extension of the intrapelvic fascia between the vagina and rectum is the rectovaginal fascia.

The muscles that elevate the anus (Levator Ani Complex), as well as the ischiococcygeus (coccygeal muscles), form the pelvic diaphragm, which maintains the tone and elevates the pelvic organs in the upper and anterior regions.

**Figure 1:**
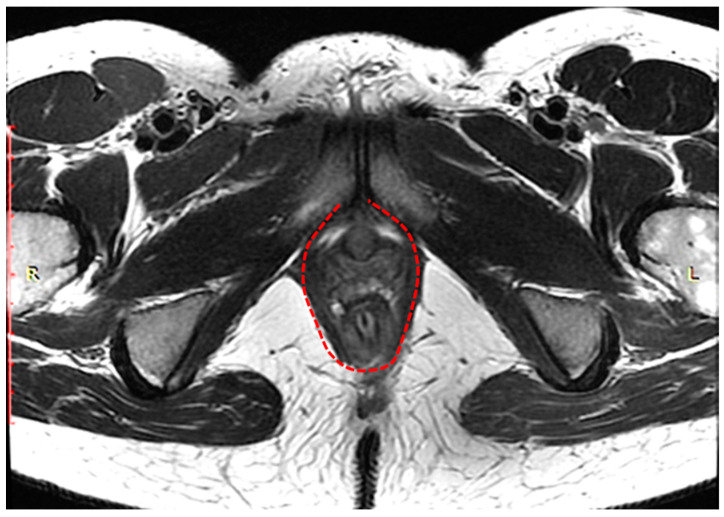
Pubo-rectal muscle U-shaped is highlighted in red, in the form of a loop covers urethra, vagina, and rectum, forming urogenital cleft.

The complex that raises the anus consists of the pubo-rectal, pubo-coccygeal, and iliopsoas-coccygeal muscles. Of these, the pubo-rectal rectus muscle (Figure 1), which branches from the pubic bone in front and covers the anorectal transition forming the urogenital cleft, holds the pelvic floor organs together and adjoins the pubic bones, preventing prolapse of these organs, and also forms the angle between the anal canal and rectum and plays a role in retaining feces [35].

The iliococcygeal muscle is oriented horizontally, forming a lower curve, and is attached to the tendon arch and coccyx, which is best visualized on the coronal projection. The Iliococcygeal muscle is derived from the upper branch of the pubic bone and attaches to the tendon arch and coccyx.

The urogenital diaphragm forms the lower parts of the pelvic floor, consisting of muscles and connective tissue that attach to the pubic symphysis, sciatic branches, and perineum. The urethra and vagina pass through the urogenital diaphragm. The muscular component of the diaphragm consists of the urethral sphincter, which surrounds the urethra, providing urinary retention, and the deep transverse perineal muscle, which provides support for the distal vagina [36].

**Figure 2:**
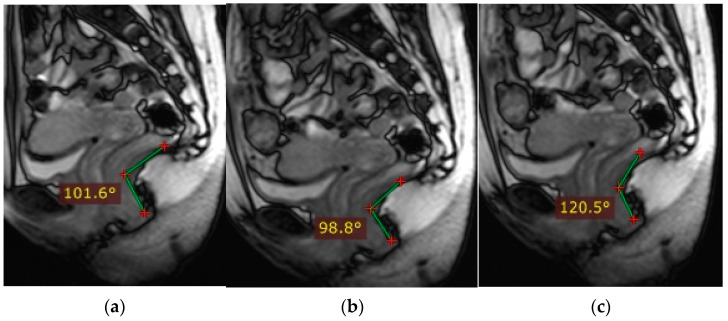
Normal indicators of pelvic organ condition. (**a**)—anorectal angle at rest, (**b**)—anorectal angle during bowel obstruction and tension of the puborectalis muscle, (**c**)—anorectal angle during relaxation of the puborectalis muscle.

Figure 2 schematically shows the landmarks in images of the pelvic organs in normal conditions: The anorectal junction is the point of intersection of the anal canal axis with the axis of the lower ampullary segment of the rectum (measured from the back of the intestine). At rest, the anorectal junction is located above or at the level of the pubococcygeal line (PCL); when pushed, it shifts 3 cm below the (PCL). The anorectal angle varies depending on the tension or relaxation of the pubic rectus muscle. At rest, it is at 90–110°, and when the pubic rectus muscle is relaxed, it opens to 103–137°; when the pubic rectus muscle is strained, it becomes sharper.

## 5. The dMRI Technique

In preparation for dMRI, the patient is asked not to eat for 4 h prior to the examination; in some cases, bowel preparation is performed to prevent motor artifacts due to bowel peristalsis. Before the study, the patient is asked to empty his bladder completely, and then 200 mL of water should be drunk to allow the bladder to partially fill during the procedure. Excessive stretching of the bladder walls may reduce the accuracy of the results due to flow artifacts from the movement of urine in an overfilled bladder and will also make it difficult to perform the commands with pressure, leading to an underestimation of bladder prolapse [37].

One hundred and twenty milliliters of sterile lubricating gel are injected into the rectum with a syringe to simulate defecation and assess complete emptying of the intestine. Next, 10–20 mL of gel is injected into the vagina. Bladder filling is not required because urine has a high signal on T2-weighted images (VVI).

The examination is usually performed on an MRI scanner with a magnetic field strength of 1.5 tesla (1.5 Tl), with the patient lying supine with a pillow under the knees. When using an MRI scanner with a magnetic field strength of 3Tl, the number of motor artifacts due to intestinal pneumatization and intestinal peristalsis increases, which complicates image assessment.

Intravenous injection of a contrasting agent is not required for MRI.

The examination is performed in the supine position using a surface coil. The table position is centered on the pubic symphysis, which allows the large-field images to capture all pelvic organs and pelvic floor muscles.

Standard T2-weighted images (T2-WI) are then obtained in three projections in the patient’s resting state. These programs assess the condition of pelvic floor support structures, including all pelvic muscles, as well as the condition of other pelvic organs.

After the pelvic floor structures and pelvic organs have been assessed, the second “dynamic” scanning step is performed, during which the patient is asked to perform a series of commands: 1—tensing the pelvic floor muscles—“pulling them in”; 2—then pushing—“pushing them out”; 3—and relaxing. To assess pelvic structural prolapse, the dynamic study is performed in the sagittal projection, positioning a single, 6–8 mm-thick slice located along the midline of the patient’s body, so that the slice field includes the pubic symphysis, sacrum, uterus, vagina, bladder, and rectum. The slice plane is scanned for 15 s, during which the patient is asked to inhale, tense the pelvic floor muscles for 2–3 s and push for 2–3 s, and then relax and breathe calmly. After that, automatic post-processing of the obtained images with the construction of a “cine-loop” is performed, which allows one to estimate the position of pelvic organs at rest and when pushing with regard to immobile pelvic bone structures (pubic symphysis and sacrum). The next step is to repeat the study with functional tests, but at the end of it, the patient is asked to empty the bowels if possible (the study is performed in a diaper) [37,38].

Almost complete evacuation of the rectal gel is considered an effective result. If this does not occur at the second attempt at emptying, subsequent attempts are made to ensure at least partial bowel emptying. In patients with partial rectal gel emptying, the degree of effort during the emptying phase is assessed by the protrusion of the anterior abdominal wall.

After the evacuation images are obtained, the examination is completed.

## 6. Image Analysis

Image interpretation begins with a review of three orthogonal T2-weighted images (T2-WI) obtained at rest to assess baseline anatomy of pelvic muscles and pelvic organ positioning. Dynamic images are then studied, assessing the position of the pelvic organs relative to the pelvic bones during different phases of the bowel and during evacuation of the gel from the colon and vagina.

**Figure 3:**
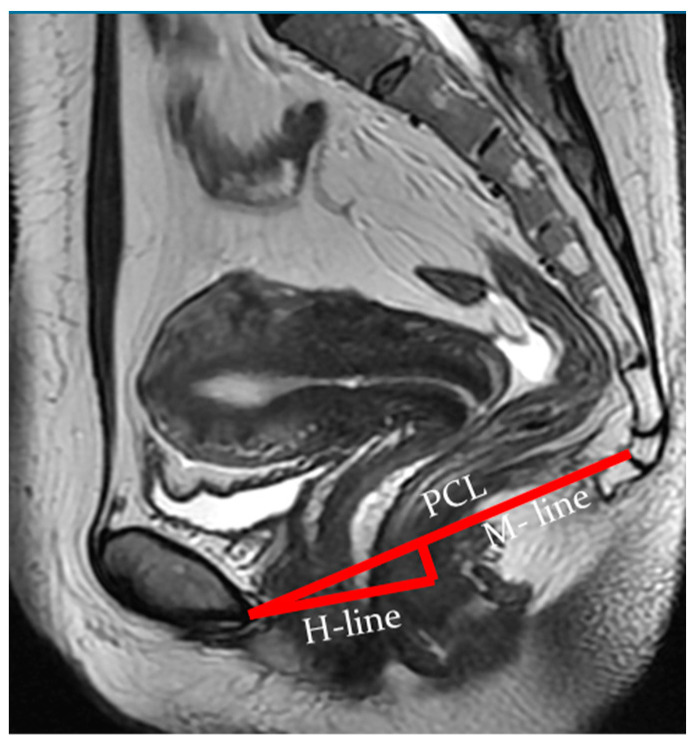
Assessment of pelvic organ parameters in relation to PCL and H and M lines.

Indicators of the condition of the pelvic organs are assessed in relation to the PCL, connecting the lower edge of the pubic symphysis with the last coccygeal junction; the H line—the line connecting the lower edge of the pubic symphysis with the posterior wall of the anorectal junction, which corresponds to the urogenital slit, and its sagittal size must not exceed 5 cm; and the M line—the perpendicular connecting the H line with the PCL, which must not exceed 2 cm (Figure 3).

The reference points of the study are:

- The base of the bladder for the anterior compartment.
- The apex of the cervix or the apex of the vagina in patients who have undergone hysterectomy for the middle compartment.
- The anorectal junction for the posterior compartment.

**Figure 4:**
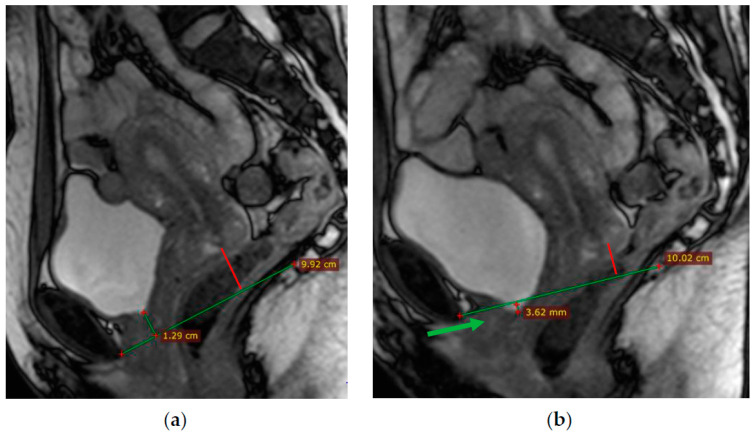
(**a**)—Normal location of the bladder floor, urethral axis, and cervix in relation to the PCL at rest—the bladder floor is located 1.3 cm above the PCL (green line) and cervix is located 2.5 cm above the PCL (red line); (**b**)—during pushing—the bladder floor shift slightly down; the urethral axis is vertical (green arrow), the urethral lumen does not gape; the cervix is 8 mm above the PCL (red line).

**Figure 5:**
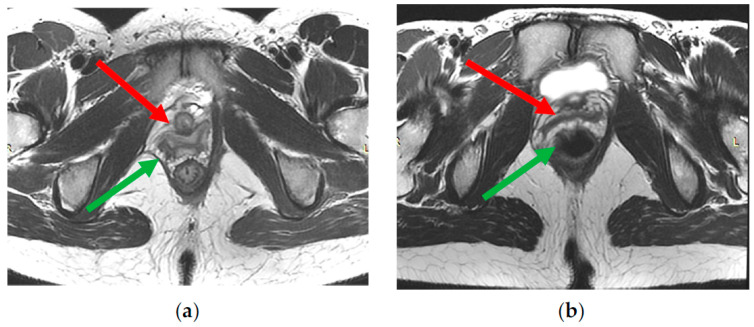
(**a**,**b**)—The signs of partial damage of right part of levator ani complex—asymmetric thinning, tortuosity of fibers course, irregular increase in muscles signal intensity on T2-WI (green arrow). Deformation of the right vaginal wall (red arrow) due to damage to right part of levator ani complex.

At rest, the bladder floor is located 2 cm above the PCL. The urethral axis is vertical at rest and may shift slightly when pushing, and the urethral lumen does not gape. At rest, the cervix is 4 cm above the PCL. When pushed, the cervix is 8 mm above the PCL with the H and M lines lengthening slightly while pushed (Figure 4 and Figure 5).

Anterior pelvic diseases that can be diagnosed using dMRI include abnormalities in pelvic floor support structures, cystocele, urethral hypermobility, and urethral sphincter insufficiency [37,38].

According to the results of the study by Rechi-Sierra K et al. comparing the clinical stage of anterior pelvic prolapse with the magnetic resonance imaging (MRI) stage, using the pubococcygeal line (PCL), midpubic line (MPL), and H line as reference lines in 42 women, it was shown that the use of the H line on MRI as reference lines has better agreement with clinical staging than PCL or MPL. The agreement of MRI with the clinical stage was assessed using the Pearson correlation for grades and the Kappa index for stages, and inter-rater reliability was assessed using the intraclass correlation coefficient (ICC) [17].

### 6.1. Distortion of Images of Supporting Structures of the Urethra

Muscles, ligaments, and fasciae are assessed by the intensity of their signals and the integrity of the MRI image. The pelvic floor structures may be weakened or damaged, covering only the anterior region or all regions, including the middle and posterior regions.

Ligaments appear as symmetrically arranged thin linear structures of low signal intensity on T2-WI. The pelvic floor muscles also have a uniform low signal intensity on T2-WI but are thicker. The signs of partial damage include asymmetric thinning, structure separation, tortuosity of fibers course, and an irregular increase in their signal intensity on T2-WI (Figure 5). A sign of complete damage may be the disruption of the integrity of a muscle or ligament fiber course and/or its fragmentation into several parts. Normally, the iliococcygeal muscle can have a different thickness depending on the state of rest or tension, which is 2.9–3.9 mm [37]. The puborectal muscle is usually 4.9–6.5 mm in size.

Damage to the levator ani complex is found in 19–28% of women after natural childbirth [38]. Lockhart M. et al. (2018) performed a pre and postpartum dMRI; the study demonstrated a relationship between a levator rupture and POP after pregnancy and delivery [39]. Other studies have confirmed the relationship between defects of this muscle and anterior pelvic prolapse [40].

According to dMRI data, regardless of prolapse status, women with levator defects have a lower pelvic organ location due to a widening of the urogenital slit [41]. H and M lines are used to estimate the size of the urogenital crevice on MRI. The H line is a representation of the anteroposterior dimension of the urogenital crevice and is usually <6 cm in length; an increase in this distance indicates a widening of the urogenital crevice, which may be the cause of pelvic organ prolapse. An increase in the H line to 6–8 cm is regarded as a mild degree of widening of the urogenital crevice, 8–10 cm is a medium degree of widening, and more than 10 cm is a severe degree.

The M line, which is the vertical distance from the PCL to the H line at the level of the anorectal junction, is usually <2 cm in size. An increase in the M line to 2–4 mm is a sign of mild posterior compartmental prolapse, 4–6 cm is a moderate degree, and more than 6 cm is a severe prolapse [41].

### 6.2. Cystocele and Hypermobility of the Urethra

**Figure 6:**
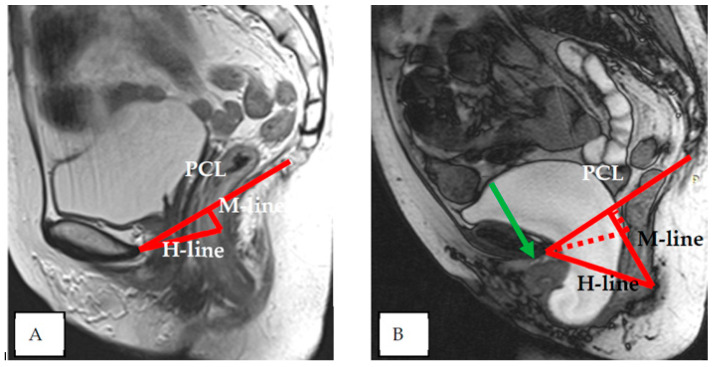
Pelvic structures at rest (**A**) and during pushing (**B**)—the bladder floor shift more than 6 cm below the PCL—severe cystocele; the urethral axis is horizontal (green arrow), the urethral lumen does not gape; the H and M lines are increased significantly—severe degree of the widening of the urogenital slit.

A cystocele, or anterior pelvic floor prolapse, is a prolapse of the base of the bladder. This pathology is usually detected during bowel movement and defecation but can also occur at rest. As the size of the cystocele increases, the size of the urogenital crevice increases due to the prolapse of part of the bladder with a deformation of the vaginal wall (Figure 6B). This is clinically manifested by a palpable bulge and can cause urinary incontinence and/or voiding dysfunction. Similar changes can develop when the pubocervical fascia and the puborectal muscle are weakened [42].

Normally, at rest, the bladder neck is above the PCL, whereas when the pelvic floor muscles are weak, the bladder neck may drop to the level of the (PCL) or even below it. During normal bowel pressure, the bladder neck is slightly downward, but not less than 1 cm from the level of the PCL. As illustrated in Figure 6, cystocele size is assessed with pressure in relation to the PCL. The 3-cm rule is used to assess the degree of cystocele: Prolapse of the bladder neck 1–3 cm below the PCL on exertion is considered to be a mild prolapse, 3–6 cm below the PCL is a moderate prolapse, and more than 6 cm is considered severe [43].

In the normal state, the urethra is positioned in the retropubic space, perpendicular to the pelvic floor on the mid-sagittal projection at rest and tension (Figure 6A). Sometimes, there may be a slight horizontal curvature of the urethra.

Patients with urethral hypermobility (UH) are characterized by a more pronounced horizontal rotation during the evacuation of contents due to insufficient urethral ligaments, which can lead to a stress type of urinary incontinence (Figure 6B). In clinical practice, UH is diagnosed by using a cotton swab when the tilt angle changes by 300 or more with urethral distension. When UH is not detected, a combined urodynamic examination is performed to assess pressure. Like the urethral test, Q-tip dMRI can detect UH when the urethral angle is increased by 300 or more. A severe cystocele can result in abnormal prolapse of the urethrovesical junction, which can mask the symptoms of urinary incontinence in the presence of concomitant UH [40].

A number of studies emphasize the importance of performing dMRI not only with bowel movements but also with obligatory defecation to assess the true size of a cystocele. For example, Arif-Tiwari H. et al. (2018) compared images with defecation and images without defecation (with only the Valsalva test) in 237 women with symptoms of prolapse. The authors showed that in many patients, the size of the cystocele increased to 3.4 cm relative to the LCL when the defecation test was performed; whereas, when the Valsalva test was performed alone, the cystocele prolapsed only 1 cm below the (PCL) [44].

In the clinical examination, a standardized assessment of POP is performed using Aa and Ba points [45,46,47]. This examination is performed in the dorsal lithotomy position and/or standing when the patient is asked to perform the Valsalva test. Point Aa is located along the anterior wall approximately 3 cm proximal to the hymen.

Point Ba is the most distal point of the anterior vaginal wall and its location ranges from −3 cm to +TVL (total vaginal length). The prolapse then progresses to stages 0 to 4. Stage 0 means no prolapse. In stage 1, the distal part is more than 1 cm proximal to the hymen. Stage 2 prolapse is within 1 cm (distal or proximal) of the hymen. Stage 3 prolapse is more than 1 cm distal to the hymen, but less than 2 cm total vaginal length. Stage 4 prolapse describes a complete eversion in which the distal part of the prolapse protrudes at least 2 cm of the total vaginal length [47,48,49].

### 6.3. Insufficiency of the Urethral Muscles

**Figure 7:**
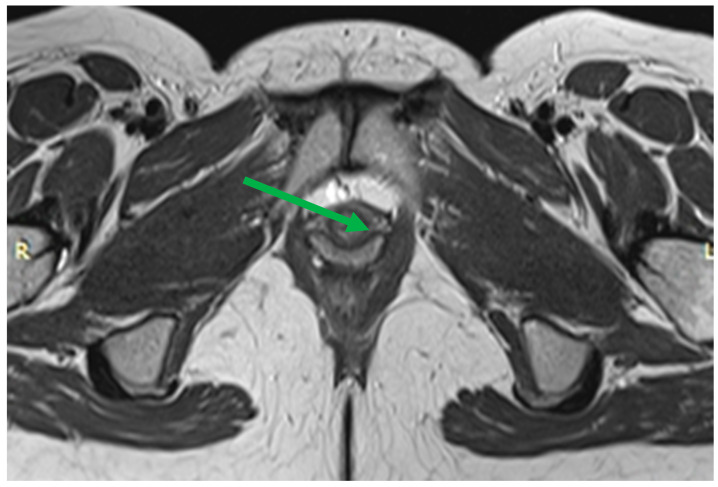
Normal urethra (arrow) on axial T2-WI: The mucosal and submucosal layers have high signal intensity, the transverse striated muscle layer is hypointense, and the middle smooth muscle layer is hyperintense. Thinning of the transverse striated urethral sphincter muscle is also associated with urinary loss [51], although the relationship is debated [52,53]. Weakening of the urethral sphincter can lead to urethral diverticulum formation (Figure 8). These changes can manifest as urethral obstruction, followed by infection of the paraurethral glands. Diverticula can vary in size, with the larger ones looking like a fluid-filled pouch surrounding the urethra. To make this diagnosis, it is sufficient to perform a normal MRI examination without performing functional tests with pressure.

Normal urethra looks like a “target” on axial T2-WI. The mucosal and submucosal layers are characterized by high signal intensity, the transverse striated muscle layer is hypointense, and the middle smooth muscle layer is hyperintense (Figure 7). Shortening of the urethra and insufficiency of its sphincter can be assessed by dMRI. Patients usually have stress urinary incontinence similar to that of UH. When the urethra is less than 3 cm in length, it can be seen to be shortened on MRI scans [50].

### 6.4. Multi-Compartmental Assessment

Diagnosis of a multicomponent lesion can be difficult, although this variant of pathology is quite common. dMRI of the pelvic organs provides visualization and functional analysis of all three parts of the pelvis and often allows the choice and correction of surgical treatment tactics [32,34].

Disorders of the mid-pelvic floor support structures can lead to uterine or vaginal prolapse. The diagnosis of mid-compartment prolapse is assessed similarly to cystocele using the “3 cm rule” [43].

**Figure 9:**
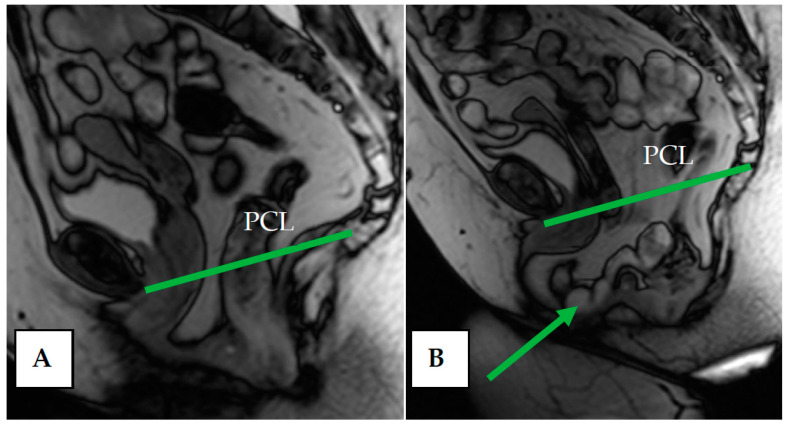
(**A**) Pelvic structures at rest. (**B**) Enterocele and peritoneocele during pushing—prolapse of the small intestine loop and mesentery between the vagina and rectum into the recto-vaginal space (arrow); atypical deepening of the Douglas space.

When the rectovaginal fascia is impaired, the posterior compartment of the pelvic organs can become prolapsed, resulting in enterocele, peritoneocele, or sigmoidocele, which are easily detected on dMRI images. In enterocele and peritoneocele, there is a prolapse of the small intestine loop into the recto-intestinal-vaginal space (Figure 9). On dMRI of enterocele, the following is noted: Location of the small intestine or mesentery between the vagina and rectum, location of the bowel loops below the PCL, dilation of the rectovaginal-vaginal septum, and atypical deepening of the Douglas space [53,54].

Posterior prolapse of the rectocele results from anterior bulging of the anterior wall of the rectum. Clinically, it is felt as a bulge on the posterior vaginal wall. Other clinical manifestations of posterior compartment organ prolapse include pelvic organ dysfunction, intussusception, and fecal incontinence.

## 7. Limitations of dMRI

A number of points limit the routine use of dMRI. For example, some authors disagree on the accuracy of MRI in the evaluation of POP, while some authors testify to high levels of sensitivity, positive predictive value, and negative predictive value of dMRI data in the diagnosis of cystocele, compared with intraoperative data. Other authors, in contrast, note the low consistency of dMRI results and clinical examination data in all three pelvic regions [55], and Ramage L. et al. (2018) demonstrated a weak correlation between dMRI and the severity of patient complaints [56]. Fauconnier A. et al. (2008) also note relatively low correlations between obtained MRI data and the results of clinical examination of patients when assessing all three parts of the pelvis [47].

A study by Rosenkrantz A. et al. (2014) showed that signs of POP are observed on dMRI in asymptomatic women, which indicates the need for further research in this area [57].

Assessment of the patient’s strain and maximal evacuation efforts during the examination should be performed by well-trained specialists. However, this is complicated in cases when the patient feels discomfort during the dynamic part of the examination. Also, the application of the method is time-consuming and requires appropriate preparation of the patient.

## 8. Conclusions

Thus, dMRI is a method of investigation, which is characterized by the high resolution of the obtained images and reproducibility. The method allows one to reveal the pathology of organs and supporting structures of all pelvic sections and is characterized by reliable correlations of the obtained results with clinical data. It is especially important when examining patients with a complicated medical history or in cases of doubtful examination results, as well as in cases of multicompartment disorders.

The use of dynamic imaging makes it possible to assess functional changes accompanying pelvic floor pathology. Of course, the dMRI method does not replace clinical assessment, but it can be used as an additional tool to assess the condition of pelvic organs and pelvic floor structures; its results allow for making an objective assessment of the condition of pelvic floor structures. This is especially important when choosing the tactics for surgical treatment of pelvic organ prolapse.

## Figures and Tables

**Figure 8:**
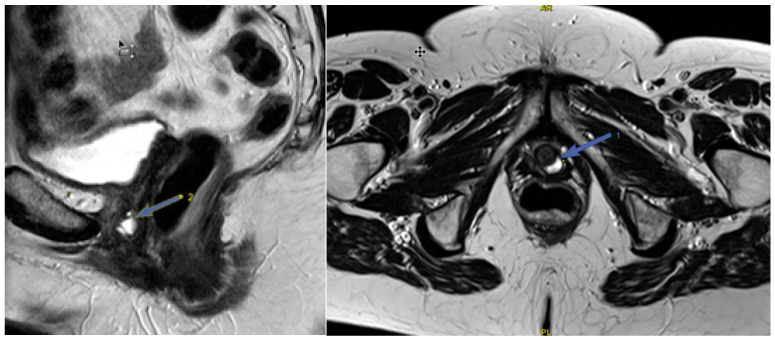
Urethral diverticulum (arrow) on sagittal and axial T2-WI: A fluid-filled pouch behind the urethra.

## Data Availability

The data presented in this study are available upon request from the corresponding author.
